# Complicações na remoção de implantes em ortopedia pediátrica

**DOI:** 10.1055/s-0045-1814113

**Published:** 2025-12-30

**Authors:** Marcela de Andrade Balsano, Heloisa Zimmermann Faggion, Alexander Cordeiro Bornhold, Weverley Rubele Valenza, Jamil Faisal Soni

**Affiliations:** 1 Hospital do Trabalhador, Curitiba, PR, Brasil; 2 Hospital Universitário Cajuru, Curitiba, PR, Brasil

**Keywords:** complicações pós-operatórias, ortopedia, pediatria, remoção de dispositivo, device removal, orthopedics, pediatrics, postoperative complications

## Abstract

**Objetivo:**

A remoção de implantes é uma prática comum na ortopedia pediátrica, apesar de seus riscos. Este estudo visa avaliar as complicações pós-operatórias da remoção de implantes em pacientes pediátricos, correlacionando-os com fatores epidemiológicos.

**Métodos:**

Estudo transversal retrospectivo, realizado em um hospital terciário, com análise de prontuários e exames de imagem entre fevereiro de 2021 e junho de 2024. Foram avaliados prontuários de pacientes menores de 18 anos, acompanhados até a alta ambulatorial. A pesquisa incluiu idade, sexo, tipo de implante, indicação da inserção e retirada, tempo de permanência do implante e complicações pós-operatórias, que foram classificadas de acordo com Clavien-Dindo.

**Resultados:**

Foram analisados 202 prontuários. A retirada de implantes foi mais comum em meninos, com média de idade de 12 anos e tempo médio de permanência de 16 meses. O principal motivo da colocação foi trauma ortopédico, e o da remoção, consolidação óssea. A taxa de complicações foi de 10% (n = 22). A remoção de placas teve a maior taxa de complicações (15%), seguida por parafusos isolados (14%), fixadores externos (12%), hastes flexíveis (10%) e fios de Kirschner (8%). As principais complicações foram retirada malsucedida (45,5%), infecção superficial (36,5%), refraturas (9%) e limitação de movimento (9%). A classificação de Clavien-Dindo revelou 45,45% de complicações tipo I, 40,9% tipo II e 13,6% tipo IIIa.

**Conclusão:**

A remoção de implantes dentro da ortopedia pediátrica não é isenta de complicações, sendo encontradas 11% neste estudo. A retirada completa malsucedida do implante, infecções superficiais e refraturas foram as mais comuns. Antes do procedimento, os riscos e benefícios envolvidos devem ser considerados, sendo necessário um consenso com familiares e cirurgiões.

## Abstract (en)

**Objective:**

Implant removal is a common practice in pediatric orthopedics, despite its risks. This study aims to evaluate postoperative complications following implant removal in pediatric patients, correlating them with epidemiological factors.

**Methods:**

Retrospective cross-sectional study, conducted in a tertiary hospital, with analysis of medical records and imaging exams from February 2021 to June 2024. Medical records of patients under 18-years-old who were followed up until outpatient discharge were evaluated. The research included age, sex, type of implant, indication for insertion and removal, time to implant removal, and postoperative complications, which were classified according to Clavien-Dindo.

**Results:**

A total of 202 medical records were analyzed. Implant removal was more common in boys, with a mean age of 12 years, and the mean time to removal was 16 months. The main reason for placement was orthopedic trauma, and for removal, bone consolidation. The complication rate was 10% (n = 22). Plate removal had the highest complication rate (15%), followed by isolated screws (14%), external fixators (12%), flexible nails (10%), and Kirschner wires (8%). The main complications were unsuccessful removal (45.5%), superficial infection (36.5%), refractures (9%), and movement limitation (9%). The Clavien-Dindo classification revealed 45.45% type I complications, 40.9% type II complications, and 13.6% type IIIa complications.

**Conclusion:**

Implant removal in pediatric orthopedics is not without complications, with 11% being found in this study. Failure to completely remove the implant, superficial infections, and refractures were the most common. Before the procedure, the risks and benefits involved should be considered and consensus should be reached among family members and surgeons.

## Introdução

A remoção dos implantes após a consolidação de uma fratura é um procedimento prevalente na prática ortopédica. 1 Estima-se que 6% de todos os procedimentos ortopédicos sejam retiradas de implantes, e na população pediátrica este índice pode chegar a 6,7%. 2

Ainda não existe uma diretriz clínica que determine a indicação e o momento da remoção dos implantes, fazendo deste tema um tópico de discussão na comunidade ortopédica. 3

Os benefícios da remoção dos implantes incluem a prevenção de sequelas biológicas e funcionais, como indução a tumores, infecção, inflamação e, se necessário, maior facilidade de realização de cirurgias reconstrutivas. 4

A remoção dos implantes não é um procedimento isento de riscos e complicações, então sua indicação deve ser discutida com os responsáveis no pré-operatório. Durante a remoção, há risco de dano de estruturas neuro vasculares, quebra de parafusos, impossibilidade de retirada completa do material, necessidade de ampliação da incisão inicial ou realização de novas, evolução com infecção superficial ou profunda e aumento do risco de refratura. 3 O risco de complicações envolvido na remoção do material de síntese relatado na literatura é em média de 10%. 5

Não existem evidências na literatura atual que apoiem ou refutem totalmente a remoção rotineira de implantes em crianças. Portanto, o estudo da retirada dos implantes é interessante devido ao seu impacto nas práticas ortopédicas e custos de serviços de saúde. 3

Este estudo objetiva analisar as complicações pós-operatórias nas remoções de implantes em pacientes pediátricos, correlacionando os fatores epidemiológicos envolvidos e tipos de implante retirados.

## Materiais e Métodos

Trata-se de um estudo transversal retrospectivo, baseado em prontuários e exames de imagem dos arquivos de um hospital terciário, entre fevereiro de 2021 e junho de 2024.

Este estudo foi submetido ao do Comitê de Ética em Pesquisa e aprovado sob o parecer substanciado número CAAE: 83355224.6.0000.5225.

Foram analisados os pacientes, menores de 18 anos, submetidos à cirurgia de remoção do implante os quais foram acompanhados até alta ambulatorial e com preenchimento do prontuário médico de forma completa, sendo a estimativa amostral de 350 pacientes.

Foram excluídos aqueles apresentando implantes para fora da pele, que passaram por retirada ambulatorial do implante, aqueles com o preenchimento do prontuário médico de forma incompleta e os que perderam seguimento pós-operatório.

As variáveis coletadas para análise foram: idade, sexo, tipo de implante utilizado, indicação cirúrgica da inserção (trauma x enfermidade ortopédica), local da inserção do implante (membro superior ou membro inferior), tempo de permanência, indicação da retirada e complicações relacionadas a retirada.

Foram consideras complicações pós-operatórias relacionadas a retiradas dos implantes neste estudo: remoção malsucedida do implante, refraturas, limitação de amplitude de movimento no período pós-operatório e infecções superficiais na ferida operatória.

Para a análise das complicações pós-operatórias encontradas utilizou-se a Classificação de Clavien-Dindo (CCD). 6 7 No tipo I, qualquer desvio do curso pós-operatório ideal sem necessidade de tratamento farmacológico ou intervenções cirúrgicas e/ou radiológicas. Pacientes tipo II necessitam tratamento farmacológico com drogas diferentes daquelas permitidas para complicações do tipo I. Tipo III exige intervenção cirúrgica e/ou radiológica, sendo (a) sem e (b) com anestesia geral. Tipo IV refere-se a complicações com risco de vida, incluindo disfunção (a) de apenas um e (b) de múltiplos órgãos. Por fim, tipo V refere-se a morte do paciente. 7

Os dados obtidos foram analisados quantitativamente através do software Microsoft Office Excel 2010 (Microsoft Corp.) para medidas de frequência absoluta e relativa. As comparações entre as variáveis foram realizadas por meio do teste de Qui-quadrado para as variáveis quantitativas. As análises estatísticas foram desenvolvidas no programa R (RStudio, 2020) sendo considerados significantes os valores de *p* < 0,05.

## Resultados

**Fig. 1:**
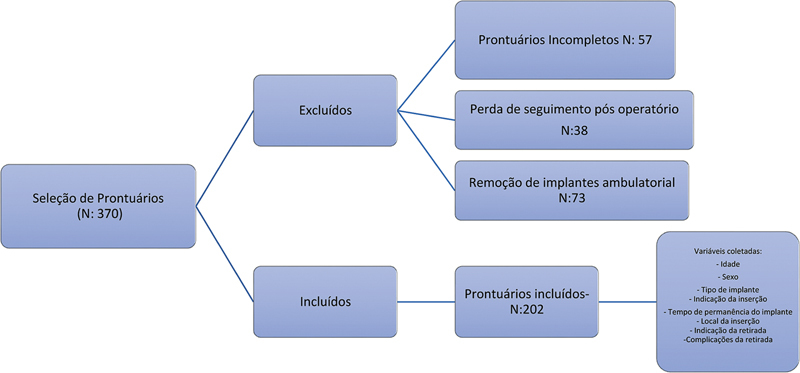
Fluxograma da seleção dos prontuários de pacientes.

Foram selecionados 370 prontuários de pacientes de 0 a 18 anos, submetidos a retirada de implantes entre fevereiro de 2021 a junho de 2024. Após a aplicação dos critérios de exclusão, restaram 202 como amostra analisada, conforme Fig. 1 .

Dos 202 pacientes da amostra, 156 (77,2%) foram do sexo masculino e 46 (22,8%) do feminino. A média de idade da amostra foi de 11,5 anos, variando de 4 a 17 anos. No sexo masculino a média de idade foi de 11,7 anos e no feminino de 10,8 anos.

O tempo médio de permanência dos implantes foi de 9,7 meses, variando de 1 a 72 meses. Na Tabela 1 , observa-se os valores de média de tempo de permanência dos implantes nos pacientes que apresentaram complicações (14,4 meses) versus os que não apresentaram (10,3 meses).

**Tabela 1:** Tempo de evolução (meses) segundo presença de complicação em 202 pacientes

Complicações	Média de evolução (meses)	DP	Valor de *p*
Não (n = 180)	10,3	9,0	0,846
Sim (n = 22)	14,4	16,7	

**Abreviação:** DP, desvio padrão.

Dos implantes retirados, foram 62 fios de Kirschner (intramedular para fixação de fratura de antebraço ou fixação trans óssea deixado sepultado subcutaneamente), 70 hastes intramedulares flexíveis, 17 fixadores externos, 41 placas e parafusos e 8 parafusos isolados. O tempo de permanência médio de cada implante até a retirada, em meses, é apresentado na Tabela 2 .

**Tabela 2:** Tempo de permanência do implante (meses) segundo material colocado em 202 pacientes

Material colocado*	Média de permanência (meses)	DP
FK (n = 62)	6,5	5,6
Fixador externo (n = 17)	4,6	3,1
Haste intramedular (n = 70)	11,0	6,8
Parafuso (n = 8)	14,3	11,5
Placa (n = 41)	18,5	15,8

**Abreviações:** DP, desvio padrão; FK, fios de Kirschner.

**Nota:** *Foram removidos pacientes com valores em mais de uma categoria de análise.

O motivo para retirada dos implantes em 83,6% (n = 169) dos pacientes foi por consolidação óssea, em 8,98% (n = 18) por incômodo e/ou proeminência relacionado ao implante, 3,46% (n = 7) por ter conseguido o alinhamento desejado do membro (pós-, hemi- ou epifisiodese), 1,98% (n = 4) por limitação de amplitude de movimento relacionada ao implante, 1,48% (n = 3) por exposição do material e 0,5% (n = 1) por infecção superficial relacionada ao implante.

A incidência de complicações foi de 11% (n = 22). Sendo que, das complicações observadas 36,5% foram casos de infecção superficial (n = 8) 9% de limitação de amplitude de movimento temporária após a retirada, que recuperaram a mobilidade no acompanhamento ambulatorial (n = 22), 9% de refraturas (n = 2) e 45,5% de retirada total malsucedida do implante (n = 10), sendo essa a complicação mais frequente. No sexo masculino tivemos 10% de complicações e 13% no feminino.

Analisando as complicações relacionadas ao tipo do implante retirado, tivemos 8% em fios de Kirschner (5/62), 12% em fixador externo (1/17), 10% em hastes intramedulares flexíveis (7/70), 14% em parafusos isolados (2/8) e 15% em placas e parafusos (7/41).

Quanto a indicação de colocação do implante, 24 foram para tratamento de uma enfermidade ortopédica (doença) onde ocorreram 17% de complicações (n = 4), 178 foram pacientes que tiveram um episódio traumático, com 10% de complicações (n = 18).

E quanto ao local do implante que foi retirado, 100 foram no membro inferior e 101 no superior, com 12 e 10% de complicações respectivamente. Os dados analisando a amostra total estão dispostos na Tabela 3 .

**Tabela 3:** Presença ou não de complicações segundo diferentes variáveis qualitativas em 202 pacientes

Variável	Categorias	Complicações (%)		Valor de *p*
		Não	Sim	
		(n = 180)	(n = 22)	
Sexo	Feminino (n = 46)	87	13	0,595
	Masculino (n = 156)	90	10	
Implante*	FK (n = 62)	92	8	0,867
	Fixador externo (n = 17)	88	12	
	Hastes flexíveis (n = 70)	90	10	
	Parafuso (n = 7)	86	14	
	Placa (n = 41)	85	15	
Motivo de colocação	Doença (n = 24)	83	17	0,307
	Trauma (n = 178)	90	10	
Localização do trauma	Membro inferior (n = 100)	88	12	0,659
	Membro superior (n = 101)	90	10	

**Abreviação:** FK, fio de Kirschner.

**Nota:** *Foram removidos pacientes com valores em mais de uma categoria de análise.

Considerando a amostra apenas dos pacientes que tiveram complicações ( Tabela 4 ), observamos que a idade média foi de 12 (6–17) anos, o tempo médio de retirada do implante foi de 16 (2–72) meses. Além disso, 50% (11–22) das complicações ocorreram nos membros superiores e 50% (11–22) nos inferiores.

**Tabela 4:** Resumo das complicações pós-operatórias após retirada de implantes agrupadas conforme características epidemiológicas avaliadas

Sexo	Idade (anos)	Material	Motivo do implante	Sítio cirúrgico	Tempo de evolução (meses)	Motivo de retirada	Complicações
F	6	Haste intramedular	Fratura diafisária de fêmur	Coxa	7	Consolidação	Infecção de FO
M	9	Haste flexível intramedular	Fratura diafisária de fêmur	Coxa	8	Infecção de FO	Restrição de ADM
M	9	Placa 2,7MM	Artrodese de hálux direito	Pé	10	Consolidação	Infecção de FO
M	9	Parafuso esponjoso 3,5MM + arruela	Pseudoartrose de côndilo lateral	Cotovelo	7	Limitação de ADM	Retirada malsucedida
F	10	Placa angulada de quadril	Luxação paralítica do quadril	Quadril	72	Exposição do material	Infecção de FO
F	10	PCD 4,5MM	Fratura de tíbia distal	Perna	12	Consolidação	Retirada malsucedida
F	11	Fixador externo	Fratura de fêmur distal	Coxa	7	Consolidação	Refratura
M	11	PCD 4,5 MM	Fratura subtrocantérica	Coxa	12	Consolidação	Retirada malsucedida
M	11	Haste intramedular	Fratura diafisária de fêmur	Coxa	7	Consolidação	Infecção de FO
M	11	FK	Fratura de antebraço	Antebraço	6	Consolidação	Infecção de FO
M	12	FK	Fratura de antebraço	Antebraço	2	Exposição do material	Infecção de FO
M	13	FK	Fratura de antebraço	Antebraço	5	Consolidação	Restrição de ADM
M	13	PCD 4,5MM	Fratura diafisária de rádio	Punho	14	Consolidação	Retirada malsucedida
M	13	Haste intramedular	Fratura diafisária de fêmur	Coxa	24	Consolidação	Retirada malsucedida
M	13	FK	Fratura de antebraço	Antebraço	6	Consolidação	Infecção de FO
F	14	Parafuso canulado 3,0	Fratura de epicôndilo medial	Cotovelo	31	Consolidação	Retirada malsucedida
M	14	PCD 4,5mm	Fratura diafisária de fêmur	Coxa	53	Consolidação	Retirada malsucedida
F	14	Haste intramedular	Fratura diafisária de úmero	Ombro	10	Consolidação	Retirada malsucedida
M	14	FK	Fratura de antebraço	Antebraço	5	Consolidação	Refratura
M	15	Haste flexível intramedular	Fratura de úmero proximal	Ombro	16	Incômodo com material	Retirada malsucedida
M	15	Placa em 8	Discrepância de MMII	Joelho	12	Alinhamento	Infecção de FO
M	17	Haste intramedular	Fratura de antebraço	Antebraço	24	Consolidação	Retirada malsucedida

**Abreviações:** ADM, amplitude de movimento; FK, fios de Kirschner; FO, ferida operatória; PCD, placa de compressão dinâmica; TEM,.

Analisando a região anatômica em que ocorreram as complicações temos: 7 em implantes retirados da coxa, 6 no antebraço, 2 no cotovelo, 2 no ombro, 1 no quadril, 1 no joelho, 1 na perna, 1 no pé e 1 no cotovelo.

Em relação ao implante utilizado temos que 31,8% (n = 7) ocorram com uso de hastes flexíveis, 31,8% (n = 7) foram com placas, 22,9% (n = 5) com fios de Kirchner, 9,0% (n = 2) com uso de parafusos isolados e 4,5% (n = 1) com uso de fixador externo.

Analisando o motivo para a retirada dos implantes observamos que 73% (n = 16) foi em decorrência de consolidação óssea, 9% (n = 2) devido exposição do implante, 4,5% (n = 1) por limitação de amplitude de movimento, 4,5% (n = 1) por incomodo com implante e 4,5% (n = 1) por infecção superficial local.

Utilizando a CCD 6 7 foram: 10 complicações do tipo I (45,45%), 9 do tipo II (40,9%) e 3 do tipo IIIa (13,6%), como demonstrado na Tabela 5 .

**Tabela 5:** Complicações pós-operatórias após retirada de implantes agrupadas conforme o sistema de classificação de Clavien-Dindo

Tipo	N (%)
I	10 (45,45%)
II	9 (40,9%)
IIIa	3 (13,6%)
IIIb	0 (0)
IV	0 (0)
V	0 (0)

## Discussão

A remoção dos implantes é um procedimento relativamente comum dentro da ortopedia pediátrica, principalmente quando há sinais de infecção, quando o implante causa algum desconforto ou quando pode alterar o crescimento ósseo. 4

Dependendo do local do implante, a permanência pode dificultar a realização de procedimentos futuros. 8 Discute-se as vantagens ou desvantagens da remoção dos implantes em crianças, portanto o estudo das possíveis complicações destes procedimentos pode auxiliar na tomada de decisão do procedimento. 4

O estudo de AlOmran et al *.* , 1 avaliou a remoção rotineira de implantes em 167 pacientes, perfazendo uma taxa de complicações de 6%. Similarmente, o estudo de Desai et al. 8 encontrou uma taxa de 9,5% de complicações após analisar 2,176 casos. Em nossa série, após a avaliação de 202 casos, encontramos uma taxa geral de complicações de 11%, compatível com a literatura.

Também segundo Desai et al., 8 a retirada dos implantes após um longo tempo da sua inserção possui um maior risco de complicações, especialmente a saída incompleta ou quebra do material. Nestas situações o implante pode estar coberto por um calo ósseo formado devido sua longa permanência, aumentando o tempo cirúrgico para sua remoção, com possível remoção incompleta, transformando-se em um procedimento mais invasivo e com maior risco de complicações. 4 9

A maior taxa de complicações de nosso estudo está associada ao retirada malsucedida dos implantes. Em nossa amostra, o tempo médio para a retirada nos casos que ocorreram complicações foi de 16 meses, estando abaixo da média encontrada na literatura.

Analisandos os implantes que tiveram sua retirada incompleta, 2 hastes flexíveis utilizadas para tratamento de fratura de úmero não saíram devido o efeito saca rolha (corkscrew), 1 no fêmur e 1 no antebraço não saíram, devido as tentativas de retirada ocorrerem aos 16 meses de pós-operatório. Entre os casos de retirada de parafusos isolados, ocorreu a quebra do parafuso em 2, permitindo apenas a retirada parcial do implante. Nos 4 casos de parafusos usados na fixação de placas, tivemos sucesso na retirada da placa, porém com quebra de parte dos parafusos, ocasionando remoção incompleta.

A colocação de implantes normalmente ocorre com abordagem minimamente invasivas. Entretanto, a cirurgia de remoção muitas vezes pode ser desafiadora, exigindo uma incisão maior que a inicial, o que pode acarretar maior índice de complicações, como as infecções em feridas pós-operatórias. 5

A infecção pós-operatória foi a nossa segunda complicação mais frequente. As infecções foram superficiais, tratadas clinicamente com uso de antibióticos, sem a necessidade de novos procedimentos cirúrgicos.

No nosso estudo, observamos 2 casos de refraturas que ocorreram após a retirada de fio de Kirschner intramedular usados para fixação do antebraço, e outra após a retirada de um fixador externo tipo Limb Reconstruction System (LRS) para tratar uma pseudartrose infectada do fêmur, com permanência de 5 e 7 meses, respectivamente. Esses casos podem estar relacionados a insuficiência do calo ósseo formado, sendo os pacientes submetidos a novos procedimentos cirúrgicos para tratamento desta complicação. Portanto, a indicação de retirada de material deve ocorrer após a formação de calo ósseo e remodelamento completo do canal medular, evitando risco de refratura após a retirada do material. 4

Scheider et al. 10 avaliou o risco de complicação de retirada de implantes dos membros superiores em contexto hospitalar, foram 449 casos encontrando uma taxa geral de 17,1% de complicações, sendo o tempo médio para remoção dos implantes foi de 23,7 meses.

O risco de complicações não parece ser semelhante em todas as regiões do corpo, com casos sendo menos comuns nas abordagens dos membros superiores. 5 No nosso estudo, observamos uma ocorrência semelhante nas retiradas dos membros superiores e inferiores, sem diferenças estatisticamente significativas entre as complicações em relação aos membros.

Lieber et al. 4 analisaram a retirada de hastes intramedulares flexíveis em 384 pacientes, encontrando uma taxa de complicações menor, de 3,1%. O rigor técnico da cirurgia inicial impacta diretamente no procedimento de remoção, aumentando complicações em implantes colocados de forma inadequada. Em nosso estudo, 4 hastes não puderam ser retiradas (2 fratura do úmero, 1 do fêmur e 1 do antebraço); também identificamos 1 deiscência de ferida; 1 infecção superficial; e 1 paciente demorou a ganhar mobilidade do joelho após retirada.

Em relação a retirada de parafusos isolados, tivermos uma taxa de complicação na amostra total de 14%. O estudo de Zimmerman et al. 9 avaliou a retirada de parafusos utilizados na fixação de fraturas de tíbia distal com desvio após 2 anos de seguimento pós-operatório comparados a casos de manutenção do implante e suas repercussões a longo prazo. Foi realizada a remoção de implantes em 17 pacientes e nenhum apresentou complicações pós-operatórias. Apesar deste achado, este estudo destaca que muitos procedimentos tiveram dificuldades e destaca que não são completamente benignos, devendo-se considerar os riscos envolvidos, sendo necessário o devido alinhamento com os familiares e/ou responsáveis. 11

Rehm et al. 12 avaliaram a retirada de implantes de fraturas diafisárias do fêmur e observou um risco de refratura ocorrendo em média 11 meses após o procedimento. Nas retiradas de placas e parafusos, não ocorreram refraturas. Tivemos 7 complicações entre 41 remoções, 3 parafusos quebrados e não retirados, 3 infecções e 1 paciente se queixava de incômodo após a retirada.

A remoção de implantes eletiva deve ser considerada observando os riscos e benefícios envolvidos com o procedimento, por conta do alto custo e potencial dano neurovascular, quebra de implante, infecção, nova ou refratura, síndrome da dor regional complexa, entre outros possíveis resultados negativos. 5 9 Portanto, deve haver consenso entre o cirurgião e os familiares e/ou responsáveis, sobre a real necessidade de indicação, bem como alertar sobre possíveis complicações que possam advir do mesmo.

Nosso estudo possui limitações, incluindo seu caráter retrospectivo apresentando uma grande heterogeneidade na amostra, com diferentes tipos de materiais, sítios cirúrgicos e tipos de complicações. A indicação para a retirada dos implantes não seguiu um padrão de escolha, sendo a preferência dos cirurgiões responsáveis um fator a ser considerado. Novos estudos multicêntricos para avaliação das complicações após retirada de implantes são essenciais para auxiliar a comunidade ortopédica na melhor tomada de decisão.

## Conclusão

A remoção de implantes dentro da ortopedia pediátrica não é isenta de complicações, sendo encontrada uma taxa de 11% neste estudo. A retirada completa malsucedida, seguido das infecções superficiais e refraturas, foram as mais comuns. A realização do procedimento deve considerar os riscos e benefícios envolvidos, sendo necessário um consenso com familiares e cirurgiões. Sugere-se a realização de estudos multicêntricos para ampliar o conhecimento sobre este tema.
